# The impact of internet adaptability on internet addiction: the serial mediation effect of meaning in life and anxiety

**DOI:** 10.3389/fpsyt.2023.1268539

**Published:** 2023-12-12

**Authors:** Weijun Wang, Shihao Ma, Xinheng Han, Xin Zhao

**Affiliations:** ^1^Key Laboratory of Adolescent Cyberpsychology and Behavior, Ministry of Education, Wuhan, China; ^2^School of Psychology, Central China Normal University, Wuhan, China; ^3^Institute of Digital Commerce, Wuhan Technology and Business University, Wuhan, China; ^4^Key Laboratory of Human Development and Mental Health of Hubei Province, Wuhan, China; ^5^Information School, The University of Sheffield, Sheffield, United Kingdom

**Keywords:** internet adaptability, internet addiction, meaning in life, anxiety, serial mediation effect

## Abstract

**Introduction:**

Previous research has demonstrated the significant role of individual characteristics in adolescent Internet addiction. In line with this, our previous research has introduced the concept of “Internet adaptability” as a potential factor that enables individuals to effectively cope with the negative consequences of Internet use. However, further investigation is required to understand the impact of Internet adaptability on problematic Internet use, including Internet addiction, as well as its associated internal psychological factors. To address this research gap, the present study aims to examine the impact of Internet adaptability on internet addiction and explore the mediating roles of meaning in life and anxiety within this relationship.

**Methods:**

A questionnaire was used to survey 2,144 adolescents from high schools in central China to investigate internet adaptability, meaning in life, anxiety, and internet addiction.

**Results:**

The results revealed a significant negative correlation between Internet adaptability and adolescent internet addiction (*r* = −0.199, *p* < 0.01). Furthermore, our results indicated that Internet adaptability negatively predicts internet addiction (*β* = −0.086, *p* < 0.001). Additionally, mediation analyses revealed that both meaning in life (*β* = −0.060, *p* < 0.001) and anxiety (*β* = −0.032, *p* < 0.01) mediate the relationship between Internet adaptability and internet addiction. Moreover, a serial mediation effect involving meaning in life and anxiety was observed between Internet adaptability and internet addiction (*β* = −0.027, *p* < 0.001).

**Conclusion:**

These findings suggest that Internet adaptability plays an important role in alleviating individual internet addiction. Our results indicate that increasing individuals’ sense of meaning in life can help reduce anxiety, thereby potentially reducing internet addiction.

## Introduction

Internet usage has become an integral aspect of young people’s daily routines According to the report by China Internet Network Information Center (CNNIC), as of June 2023, there were 1.079 billion Internet users in China. Notably, 3.8% of them were under the age of 10, while 13.9% fell within the 10–19 age group, collectively constituting 62.1% of the overall youth population in China (1). However, with the growth of Internet penetration, there is a concurrent increase in psychological problems arising from maladaptation to the Internet. Among these challenges, Internet addiction has been a significant concern for scholars (2, 3). Internet addiction can be likened to a psychotropic drug that individuals often use as an escape from reality (4). In adolescence it can be a real syndrome: it affects boys and girls who cannot do without it and, deprived of the web, feel a strong discomfort that they cannot alleviate in any other way (5). A recent study showed that 9% of adolescents, with an average age of 14.53, exhibited high levels of internet addiction, and 11% showed overuse of online games (6). Internet addiction can have severe detrimental effects on an individual’s social and physical well-being (7), including an increased risk of depression and anxiety (8, 9), problematic behaviors (6), poorer academic performance (10), and severe sleep disorders (11), and so on. As a result, researchers have investigated various internal and external factors that influence internet addiction and have aimed to uncover its psychological mechanisms in order to develop effective interventions (7, 12–14).

Researchers in the field of positive psychology have begun to investigate approaches to minimize the adverse effects of internet addiction by maximizing the potential, motivation, and abilities of young individuals (15, 16). For example, a study involving 3,360 students in grades 5–9 revealed a bidirectional predictive relationship between positive youth development and internet addiction over multiple time points.

Enhancing positive youth development can reduce the risk of internet addiction and promote the positive development of adolescents (17). In addition, other studies have pointed out that positive psychological attributes such as school adaptation status (18), self-concept clarity, core self-evaluation and social adaption (19), career adaptability (20) can have a mitigating effect on Internet addiction. These findings suggest that that adaptability may serve as one of the protective factors against individual Internet addiction. It is within this context that the concept of Internet adaptability has emerged. Internet adaptability, as a core element of Internet adaptability, refers to the individual characteristics that facilitate maintaining a harmonious relationship with the online environment for personal survival and development (21, 22). Internet adaptability plays a pivotal role in determining the extent of internet adaptability among adolescents, permeating every stage of their engagement with the online world (22).

### The relationship between internet adaptability and internet addiction

Internet adaptability serves as a crucial indicator of an individual’s ability to positively adjust to the online environment (22). Social adaptation theory suggests that the development of adolescents in social life is influenced by the dynamic equilibrium between individuals and their environment (23). This dynamic balance can manifest as positive or negative behavioral outcomes (21). Similarly, internet adaptability manifests itself in two types: positive and negative adaptation. Negative adaptation is characterized by problematic internet behaviors (e.g., cyberbullying, online aggression) and an emphasis on internet interpersonal relationships (e.g., engaging in excessive internet socializing and placing a high value on online interpersonal relationships). Positive adaptation manifests primarily in the form of responsible internet usage, including adherence to internet norms, rational expression, and the rejection of harmful online information (21). Individuals with low Internet adaptability tend to exhibit more negative adaptations towards Internet use, such as poor interpersonal orientation on online platforms and problematic internet behaviors (21).

Moreover, the process of internet adaptability encompasses three stages: preparation, adaptation, and maintenance. During the preparation stage, individuals must develop a proper attitude towards the Internet, acquiring the necessary knowledge and skills. In the adaptation stage, individuals need to cultivate a sense of control over the Internet, enhancing their self-efficacy, and improving their adaptability to online platforms. Finally, in the maintenance stage, individuals should maintain a sense of control, enhancing their psychological resilience, and demonstrating proactive engagement with the Internet (22). Individuals with adequate internet adaptability tend to utilize internet resources in a scientific and responsible manner, harnessing the maximum positive benefits. Conversely, individuals with inadequate internet adaptability may exhibit negative internet behaviors, such as addiction towards online socialization.

According to self-determination theory, individuals must satisfy three fundamental psychological needs – autonomy, competence, and relatedness – in order to experience positive growth, integration, and development (24). When individuals are compelled to pursue goals or engage in behaviors without any alternative options, it means that they are not free to choose to engage in certain activities according to their heart’s desires, and that their need for autonomy is not being met, which further prevents the individual from satisfying the need for competence in controlling the environment and experiencing a sense of competence as a result of the need for competence. Self-regulation theory proposes that individuals employ various strategies to guide their behavior and emotions, ensuring the attainment of their goals (25). Therefore, in order to regain the satisfaction of their autonomy needs, individuals are more likely to engage in negative self-regulatory strategies (26). These may include seeking substitutes such as online games and short videos to satisfy their impaired basic psychological needs, or engaging in out-of-control compensatory behaviors such as Internet addiction to make up for this impaired satisfaction (27). Individuals who possess high levels of Internet adaptability tend to demonstrate positive attitudes towards the Internet. They also experience a greater sense of autonomy, exhibit a strong internal drive to explore the online world, and actively engage with the digital environment (23, 28). In contrast, individuals with low levels of Internet adaptability may not feel sufficiently satisfied with their autonomy needs in pursuing their goals (29). Consequently, they may resort to negative regulation strategies, such as stress avoidance, problem denial, procrastination, and even engaging in addictive behaviors like substance abuse or internet addiction (30, 31). The satisfaction of the asic psychological needs has been found to be a significant negative predictor of adolescents’ problematic Internet use behaviors and online game addiction (32). Therefore, Internet adaptability can be considered a potential predictor of Internet addiction. Building upon this understanding, our study puts forth the following hypothesis.

*H1:* Internet adaptability has a negative predictive effect on Internet addiction.

### The mediation effect of meaning in life

Meaning in life refers to the coherence and purpose individuals experience while finding significance and value in the world (33). Self-determination theory further suggests that consistently meeting individuals’ autonomy, competence, and relationship needs is essential for them to experience a higher sense of meaning in life (24). When individuals have their basic psychological needs met, their pursuit of goals becomes more meaningful and dynamic (34), leading to an increase in intrinsic motivation and a greater sense of meaning in life (35).

At the same time, when individuals perceive their online exploration as self-determined, it fulfills their need for autonomy. The satisfaction of basic needs makes individuals more likely to be well-adjusted. Those with greater internet adaptability tend to have a stronger intrinsic motivation to explore and actively participate in the online environment, thereby experiencing an enhanced sense of well-being and generating meaningful behavior (22). Research has also emphasized that adjustment is an important factor influencing students’ subjective well-being (36). It has been noted that adolescents’ level of school adjustment positively predicts life satisfaction (37), and that both well-being and life satisfaction are related to meaning in life (38). Therefore internet adaptability may be predictive of life meaning. Furthermore, Baumeister has suggested that the need for self-efficacy and control over the environment aligns with the need for competence (39). Individuals who are highly adaptable to the internet exhibit higher levels of internet self-efficacy and control (22). This increased self-efficacy and control fulfills their need for competence, which, in turn, contributes to a sense of purpose and meaning in their lives. It has also been shown that individual resilience positively predicts self-efficacy (40), and that self-efficacy has a positive correlation with meaning in life (41, 42). As a result, these individuals are more actively engaged in exploration and meaningful pursuits in the online environment. Finally, individuals with high levels of internet adaptability excel in adapting to online interpersonal interactions, enabling them to connect with others in a positive, rational, and inclusive manner, satisfying their relational needs and enhancing their experience of meaning in life. Therefore, individuals with high levels of Internet adaptability are more likely to fulfill their basic psychological needs and derive greater meaning in life through their own traits.

Furthermore, a high level of meaning in life may serve as a protective factor against Internet addiction. Individuals who possess a strong sense of self-identity and intrinsic motivation for finding meaning in life are more capable of exercising self-control and self-regulation to prevent Internet addiction (43). Conversely, individuals lacking a sense of identity and intrinsic motivation for finding meaning in life are more prone to seeking external stimulation and gratification, leading to the development of undesirable behaviors such as Internet addiction (44). Empirical research has consistently demonstrated a negative association between the sense of meaning in life and problematic Internet use (45, 46). Therefore, this study proposes the following hypothesis:

*H2:* Internet adaptability negatively predicts Internet addiction through the mediation of meaning in life.

### The mediation effect of anxiety

According to self-determination theory, individuals who have their basic needs satisfied tend to experience a higher sense of meaning in life and exhibit greater self-determination. Conversely, unsatisfied basic needs can undermine an individual’s self-determination, resulting in feelings of dissatisfaction and negative emotions such as anxiety and depression. These, in turn, can contribute to problematic behaviors (24, 47, 48). Therefore, the satisfaction of basic needs serves as a protective factor against the development of negative psychological problems, helping to alleviate discomfort and reduce anxiety levels (49). For example, research has evidenced that the satisfaction of basic psychological needs predicts anxiety and depression levels in high school students (50). Consequently, individuals may experience heightened anxiety and a diminished sense of self-determination due to their failure to meet the basic three psychological needs. Individuals with lower levels of internet adaptability tended to have lower levels of Internet self-efficacy, Internet control, and online interpersonal adaptability, and adolescents’ self-efficacy, interpersonal relationships, and self-control, which are all correlated with the sense of anxiety (51–53). Therefore, internet adaptability may predict of anxiety.

Davis proposed that pathological internet use behavior is influenced by a combination of individual psychopathogenic factors, stressors, and situational factors. Anxiety is considered as one of the key psychopathogenic factors that contribute to internet addiction (54). Chronically anxious individuals may turn to excessive internet use as a way to uphold their self-worth and evade fear, thereby reinforcing their internet-related behaviors and potentially leading to prolonged internet overuse (55). Furthermore, studies have indicated that anxiety serves as a risk factor for Internet Addiction (56, 57). Based on the literature above, our study proposes the following hypothesis:

*H3:* Internet adaptability negatively predicts internet addiction through the mediation of anxiety.

### The chain mediation effect of meaning in life and anxiety

Positive psychology, such as self-determination theory, emphasizes the significance of finding meaning in life. When individuals experience a sense of meaning and fulfillment, they are likely to experience reduced levels of anxiety and other negative emotions. Conversely, a lack of meaning or an inability to find meaning in life may contribute to feelings of hopelessness and helplessness, leading to anxiety and other psychological issues (57). Therefore, the absence of meaning in life can be considered an important factor leading to the development of anxiety (46, 56, 58). Numerous studies have demonstrated a negative relationship between meaning in life and negative emotions, including anxiety. Specifically, meaning in life has been found to be a significant predictor of anxiety (59).

According to self-determination theory, Internet adaptability plays a crucial role in fulfilling the need for competence, which is one of the three basic psychological needs. Individuals with high levels of internet adaptability are more likely to satisfy these needs, contributing to increased intrinsic motivation and meaning in life, as well as reduced anxiety. Consequently, they are less prone to internet addiction due to their increased sense of internet efficacy, control, and ability to discern internet information. Conversely, individuals with low-level internet adaptability may struggle to fulfill their autonomy, competence, and relatedness needs due to a lack of internet literacy. This can result in negative emotions such as anxiety and difficulties in finding meaning in life, which can lead to the development of internet addiction (43). In an effort to alleviate anxiety, individuals may turn to the internet as a means of rediscovering meaning in life to develop a sense of achievement and control. However, individuals with lower levels of internet control, information literacy, and internet coping strategies may become overwhelmed by the internet and develop an addiction while seeking solace and meaning (44). It is important to note that while anxious individuals may use the internet to seek meaning in life, it does not guarantee an effective pathway to experiencing a genuine sense of meaning. Instead, it often leads to internet addiction. According to the research conducted by Przybylski et al. (60), players who actively pursue meaning and purpose in online games can experience a sense of achievement and control within the virtual world. However, this pursuit also carries the risk of developing an online game addiction. Therefore, based on the aforementioned findings, our study proposes the following hypothesis:

*H4:* Internet adaptability negatively predicts internet addiction through the serial mediation of meaning in life and anxiety.

In summary, this study aims to investigate the influence of Internet adaptability on internet addiction based on self-determination theory and self-regulation theory.

**Figure 1:**
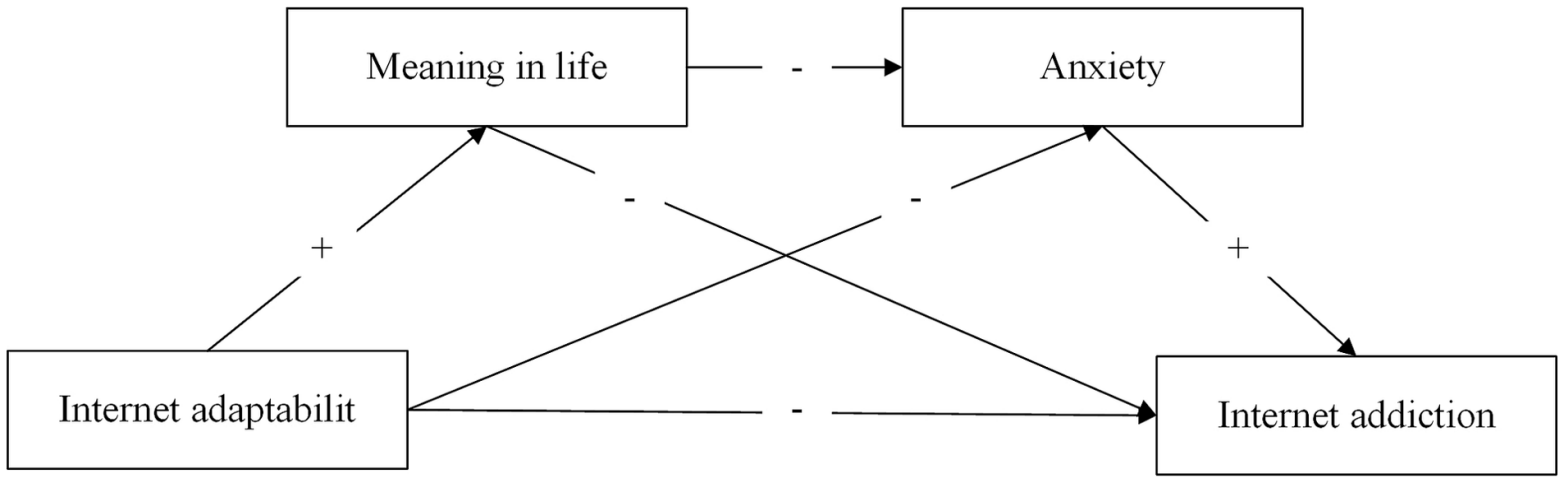
Conceptual model.

Furthermore, to examine the underlying mechanism of Internet adaptability on Internet addiction, this study incorporates two crucial psychological factors, namely meaning in life and anxiety (see Figure 1) to provide a theoretical basis for understanding and addressing adolescent Internet-related problematic behaviors and promoting healthy mental well-being.

## Materials and methods

### Participants

The researchers employed a stratified cluster sampling method to administer questionnaires in central China. The survey targeted high school students. According to the sample content estimation method proposed by Kendall, the sample content is typically recommended to be 20 times the number of independent variables (61). In this study, the Internet Adaptability Questionnaire comprises 8 primary dimensions and a total of 39 items; Meaning in Life Questionnaire comprises 2 primary dimensions and a total of 10 items; Anxiety Scale comprises 5 item; Internet Addiction Questionnaire comprises 8 item. Therefore, this study requires a sample size of at least (39 + 10 + 5 + 8) × 20 = 1,240. Taking into account the potential rate of invalid questionnaire responses, the sample size for our study should be no less than 1,500 cases. A total of 2,300 questionnaires were distributed for the subsequent formal administration. After the screening, a total of 2,144 (93%) valid questionnaires remained, including with 779 males (36.3%) and 1,365 females (63.7%). The average age of the participants was 16.11 ± 0.86 years old.

### Measurement

#### Internet adaptability questionnaire

In this study, the Internet adaptability Questionnaire was utilized to assess the Internet adaptability of adolescents (28). The questionnaire comprises 8 primary dimensions with a total of 39 items including: sense of network control (e.g., “I can make good use of the internet to serve me in my life”), network interpersonal adaptation (e.g., “I met more like-minded people online”), Internet information searching (e.g., “Words, pictures or videos of interest, I have a way to search for them online”), Internet information protection (e.g., “I will use different passwords for different accounts”), Internet positive coping (e.g., “When things get tough in networking, I adjust my emotions to accept it”), Internet learning adaptation (e.g., “Learning knowledge online has become a habit for me”), Internet self-efficacy (e.g., “I am confident that I will be able to master the latest web technologies in a relatively short period of time”), Internet curiosity (e.g., “Learning a new web technology often excites me”). Participants rated using a 6-point Likert scale ranging from “1 = strongly disagree” to “6 = strongly agree.” The Cronbach’s α coefficient for this questionnaire is 0.958, indicating strong reliability.

#### Meaning in life questionnaire

The Meaning in Life Questionnaire (62) was employed in this study to assess the meaning in life of adolescents, adapted from Steger et al. (63). The questionnaire comprises 2 primary dimensions with a total of 10 items including: Presence of Meaning (e.g., “I have discovered a life goal that satisfies me”) and Search for Meaning (e.g., “I’m looking for a purpose or mission in my life”). Participants rated their agreement using a Likert scale ranging from “1 = strongly disagree” to “7 = strongly agree.” The second item is reverse scored. The Cronbach’s α coefficient for this scale was calculated as 0.879, indicating strong reliability.

#### Anxiety scale

The short version of the Spielberger Anxiety Scale (64), was employed in this study to assess the state of anxiety among adolescents. The questionnaire comprises 5 items (e.g., “For the last month, I’ve felt distracted”; “I feel scared”; “I felt panicked inside”; “I felt terrified”; “I feel confused and muddled”), rated on a Likert scale ranging from “1 = not at all” to “4 = very much.” The Cronbach’s α coefficient for this scale was calculated as 0.93, indicating strong reliability.

#### Internet addiction questionnaire

The Internet Addiction Diagnostic Questionnaire was employed in this study to assess internet addiction among adolescents (65). The Chinese-translated version of the questionnaire was administered (66). It comprises 8 items (e.g., “I feel like I need to spend more time on the internet to be fulfilled”; “When I attempt to reduce or stop using the Internet, I feel frustrated, depressed or easily irritable”; “I often spend more time online than I originally planned”), with responses measured on a Likert scale ranging from “1 = strongly disagree” to “5 = strongly agree.” The scale demonstrated strong reliability, as indicated by a Cronbach’s α coefficient of 0.911.

### Procedure and data processing

Descriptive statistical analysis was conducted using SPSS 25.0 in this study. The AMOS 23.0 software was utilized for common method bias testing and was used to analyze the direct effect of Internet-adaptation on Internet addiction and the mediating effect of meaning in life and anxiety in the relationship between Internet-adaptation and adolescent Internet addiction.

## Results

### Control and test of common method bias

To mitigate the potential common method bias, specific measures were implemented based on previous research (67). Participants were assured of complete anonymity when responding to the questionnaire, and certain items were reverse coded to minimize response bias. Additionally, Harman’s single-factor test was conducted to assess the presence of common method bias during data collection. The analysis revealed that 11 factors had eigenvalues greater than 1, with the first factor accounting for 29.161% of the total variance, which was below the critical threshold of 40% (67). Thus, no significant common method bias was detected in the study.

### Descriptive statistics and correlations

The study employed SPSS 25.0 to perform descriptive statistical analysis on the variables, and the findings are presented in Table 1. Internet adaptability exhibited a significant positive correlation with meaning in life and a significant negative correlation with anxiety and internet addiction.

**Table 1:** Descriptive statistics and correlations among variables.

	*M ± SD*	1	2	3	4
1. Internet adaptability	4.181 ± 0.804	1			
2. Meaning in life	4.704 ± 1.069	0.495^**^	1		
3. Anxiety	1.835 ± 0.771	−0.147^**^	−0.175^**^	1	
4. Internet addiction	2.331 ± 0.827	−0.199^**^	−0.227^**^	0.422^**^	1

^*^p < 0.05, ^**^p < 0.01, ^***^p < 0.001.

### Serial mediation of meaning in life and anxiety

We first analyzed the variable normality and outlier data. In this analysis, Internet adaptability, meaning of life, and anxiety were used as independent variables, and Internet addiction was used as the dependent variable. The results showed that the variables satisfied normality and no significant outlier data were observed (see Appendix section).

**Figure 2:**
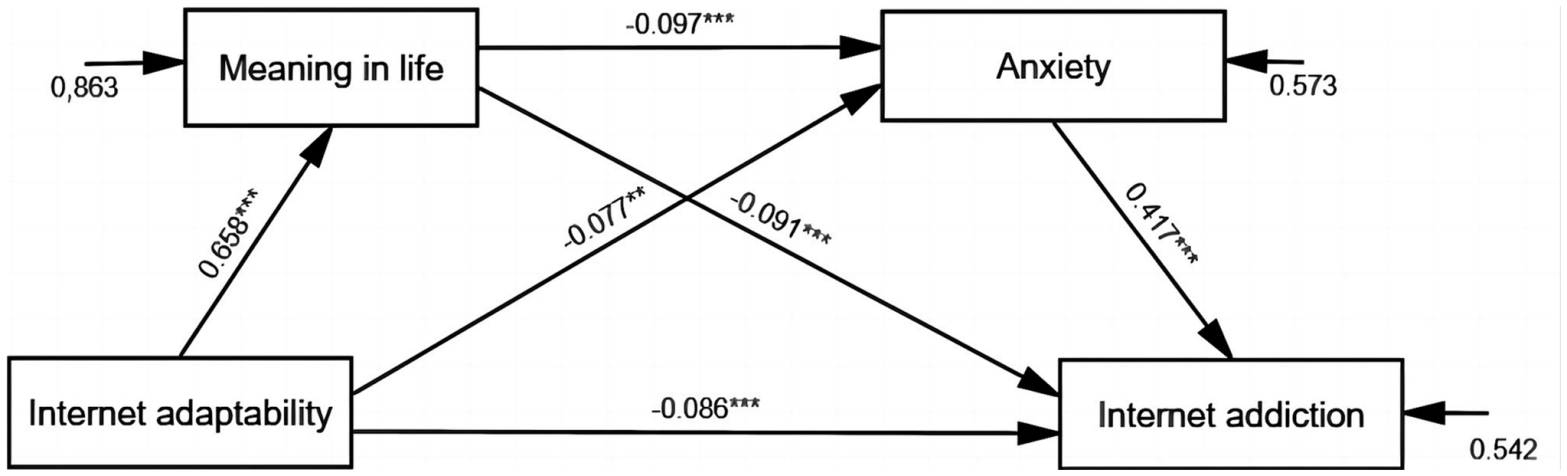
Serial mediation model. *p < 0.05, **p < 0.01, ***p < 0.001; 0.863, 0.573, 0.542 are residuals.

To examine the chain mediating effect of meaning in life and anxiety between Internet adaptability and Internet addiction, a mediation analysis was conducted using the AMOS 23.0. The maximum likelihood estimation method was employed to test for the chain mediation effect, using a bias-corrected bootstrap sample (5,000 bootstrap samples) with 95% confidence intervals. In this analysis, Internet adaptability served as the independent variable (X), Internet addiction as the dependent variable (Y), and meaning in life and anxiety as the mediating variables (M1 and M2), respectively. Model fitting was assessed using the established criteria of CFI, IFI, and GFI values ≥0.9 indicating an acceptable fit (57). The model showed a strong fit (CFI = 1, IFI = 1, GFI = 1). The results of the mediation analysis revealed significant mediation effects of meaning in life and anxiety in the relationship between Internet adaptability and Internet addiction. Specifically, three indirect paths were identified (see Figure 2).

In terms of the direct effect, our results provide evidence that internet adaptability significantly predicts internet addiction. Turning to the indirect effects, the total indirect effect was found to be significant (Table 2). Specifically, meaning in life served as a mediator [−0.060, *p* < 0.001, CI (−0.087, −0.034)] in the pathway of Internet adaptability → meaning in life → Internet addiction (X → M1 → Y). Anxiety played a mediating role [*β* = −0.032, *p* < 0.01, CI (−0.054, −0.011)] in the pathway of Internet adaptability → anxiety → Internet addiction (X → M2 → Y). Furthermore, a serial mediation effect of meaning in life and anxiety was observed [*β* = −0.027, *p* < 0.001, CI (−0.039, −0.015)] in the pathway of Internet adaptability → meaning in life → anxiety → Internet addiction (X → M1 → M2 → Y).

**Table 2:** Indirect effects of meaning in life and anxiety.

	*β*	BootSE	BootLLCI	BootULCI	Ratio of total effects (%)
Total indirect	−0.119	0.025	−0.252	−0.156	58
X → M1 → Y	−0.060	0.014	−0.087	−0.034	29
X → M2 → Y	−0.032	0.011	−0.054	−0.011	16
X → M1 → M2 → Y	−0.027	0.006	−0.039	−0.015	13

X, Internet adaptability; Y, Internet Addiction; M1, Meaning in Life; M2, Anxiety.

## Discussion

### The relationship between internet-adaptability and internet addiction

This study aimed to examine the influence of Internet adaptability on adolescent internet addiction behavior and investigate its underlying psychological mechanisms, drawing on the self-determination theory and the self-regulation theory. The findings revealed a significant negative correlation between Internet adaptability and internet addiction among adolescents, supporting *H1*. These results align with previous research highlighting the widespread issue of internet addiction among adolescents and the overall lack of social adaptation abilities in this population (68).

Internet adaptability is an essential factor influencing an individual’s social adaptation in the online environment (21) and serves as a fundamental requirement for healthy Internet engagement, the proper utilization of internet resources, and the positive outcomes derived from online activities. Individuals with high levels of Internet adaptability exhibit enhanced skills in information search and identification, enabling effective and meaningful learning experiences through the Internet. Additionally, their adept utilization of positive coping strategies and effective internet control mechanisms facilitate proactive adaptation to the online environment and serve as protective factors against internet addiction. Therefore, it is crucial to prioritize the promotion of positive adolescent development in the online space and foster the enhancement of their Internet adaptability. By doing so, we can facilitate their healthy and responsible use of the internet, encourage scientific exploration of Internet resources, and protect them against Internet addiction.

### The mediation effects of meaning in life and anxiety in the internet adaptability and internet addiction

The findings of this study indicate that meaning in life plays a partial mediating role in the relationship between Internet adaptability and Internet addiction. Internet adaptability not only has a direct negative impact on Internet addiction but also has an indirect impact on Internet addiction through its association with meaning in life, providing support for *H2*. Consistent with prior research (45, 46, 58), a higher sense of meaning in life serves as a protective factor against Internet addiction among adolescents. Drawing on self-determination theory, individuals who fulfill their autonomy, competence, and relatedness needs are more likely to experience a greater sense of meaning in life (24, 35). The extent to which these needs are satisfied largely depends on the environmental factors surrounding individuals (69). In the online context, individuals with high levels of Internet adaptability exhibit elevated levels of Internet proactivity, Internet self-efficacy, Internet control, and Internet interpersonal adaptation, enabling them to meet their basic psychological needs and cultivate a greater sense of meaning in life (35). Furthermore, individuals who experience an increased sense of meaning in life are often actively engaged in pursuing personal goals and values, rather than succumbing to internet addiction.

Furthermore, anxiety also serves as a partial mediator in the relationship between Internet adaptability and Internet addiction. Internet adaptability not only has a direct negative impact on Internet addiction but also has an indirect impact on Internet addiction through its association with anxiety, providing support for *H3*. Drawing on self-determination theory, individuals with low levels of Internet adaptability often exhibit a lower level of Internet literacy, which in turn hinders their ability to fulfill their autonomy, competence, and relatedness needs, leading to the emergence of negative emotions, such as anxiety (45, 47, 58). Furthermore, individuals facing negative emotions tend to employ negative self-regulation strategies that excessively rely on the internet as a means of evading real-life responsibilities and obligations, as a way to alleviate their inner anxiety (70–72). However, these negative self-regulation strategies ultimately contribute to the development of internet addiction.

Based on the aforementioned findings, we have found that meaning in life and anxiety both independently mediate the relationship between Internet adaptability and Internet addiction. Moreover, the chain mediating analysis has confirmed the presence of a chain mediating effect between Internet adaptability and Internet addiction, thereby supporting *H4*. Consistent with previous research, meaning in life has a significant negative predictive effect on individuals’ anxiety levels (57). Individuals with high levels of Internet adaptability are more likely to experience higher levels of intrinsic motivation and meaning in life as they can better fulfill their autonomy, competence, and relatedness needs in the online environment. On the other hand, individuals with low levels of Internet adaptability may struggle to meet these basic psychological needs, resulting in lower levels of meaning in life and extrinsic motivation, which in turn contribute to negative emotions such as anxiety. The self-regulation theory suggests that when adolescents are more influenced by extrinsic motivation rather than intrinsic motivation, they may resort to negative coping strategies (26). For instance, they may divert their attention from real-world responsibilities to the internet, seeking a sense of control in the virtual world to escape problems in their real world. This behavior can ultimately lead to Internet addiction. Therefore, in addition to the direct negative relationship that predicts internet addiction, Internet adaptability can indirectly predict internet addiction through the mediating effects of meaning in life and anxiety.

### Implications and limitations

This study examines the role of Internet adaptability in adolescent Internet addiction and the mediating role of meaning in life and anxiety. The results emphasize the significance of considering multiple factors that contribute to internet addiction, going beyond social support and family environment, and acknowledging the role of Internet adaptability. Promoting Internet adaptability offers multiple benefits, including enhancing the sense of meaning in life, reducing anxiety, and decreasing problematic behaviors on the Internet, such as excessive use of the Internet, game addiction, and so on. Therefore, the findings of this study can inform the design of intervention programs focused on Internet adaptation education for adolescents. These programs can aim to assist adolescents in setting personal goals and values, thereby increasing their sense of meaning in life, and reducing anxiety, ultimately helping to prevent internet addiction and similar issues associated with the negative effects of the Internet.

This study has several limitations. Firstly, the cross-sectional design of the study prevents us from establishing a causal relationship between internet adaptability and internet addiction. Future research could employ longitudinal designs to examine the causal nature of the relationship between Internet adaptability and Internet addiction. Secondly, meaning in life can be categorized into two distinct types: the search for meaning and the presence of meaning. Differentiating between these types can provide further insights into the specific roles they play in the relationship between internet adaptability and internet addiction. Future studies can explore the unique contributions of each type of meaning. Thirdly, the collection of demographic variables in this study was relatively limited, which may not fully capture the characteristics of internet adaptability. Future research can incorporate additional demographic variables to better understand the performance characteristics of internet adaptability. Lastly, the participants in this study were predominantly high school students, limiting the generalizability of the findings. Future research should include a more diverse sample to enhance the representativeness and broaden the applicability of the results.

## Conclusion

This study employed a stratified cluster sampling method to select a sample of 2,144 Chinese adolescents, and conducted a questionnaire survey to reveal the predictive role of internet adaptability in internet addiction, as well as the mediating roles of meaning in life and anxiety. Specifically, internet adaptability was found to have a negative predictive effect on internet addiction, with meaning in life and anxiety serving as separate and chain mediators, respectively. These findings suggest that internet adaptability can directly alleviate internet addiction in adolescents. Moreover, it can indirectly reduce their level of Internet addiction by enhancing their meaning of life or alleviating anxiety. At the same time, internet adaptability can also reduce internet addiction by acting as an anxiety reliever through enhancing the meaning of life. Overall, this study elucidates protective factors and mechanisms mitigating internet addiction risk, offering insights to guide development of evidence-based strategies grounded in positive psychology.

## Data availability statement

The raw data supporting the conclusions of this article will be made available by the authors, without undue reservation.

## Ethics statement

The studies involving humans were approved by Central China Normal University Human Ethics Committee. The studies were conducted in accordance with the local legislation and institutional requirements. Written informed consent for participation in this study was provided by the participants' legal guardians/next of kin.

## Author contributions

WW: Conceptualization, Formal analysis, Funding acquisition, Project administration, Resources, Supervision, Writing – review & editing. XH: Conceptualization, Data curation, Methodology, Writing – original draft. SM: Conceptualization, Data curation, Methodology, Writing – review & editing. XZ: Conceptualization, Formal analysis, Funding acquisition, Writing – review & editing.

## Figures and Tables

**Figure A1:**
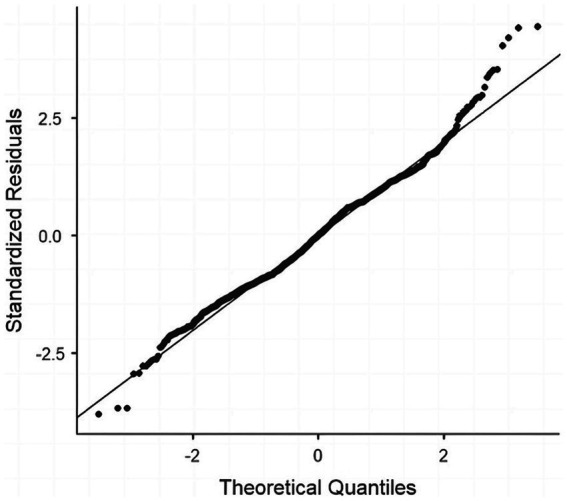
Q-Q plot.

## Appendix

### Normal distribution analysis

The skewness and kurtosis tests of each variable are as follows:

**Table A1:** Skewness and kurtosisi test.

	Skewness	Kurtosis
Internet adaptability	0.064	0.233
Meaning in life	0.174	0.305
Anxiety	0.976	0.800
Internet addiction	0.178	−0.334

The results show that skewness and kurtosis were less than ± 2, this means that all variables follow the normal distribution.

### Multivariate normal distribution analysis

The multivariate normal distribution analysis are as follows:

Among them, the independent variable includes internet adaptability, meaning in life, anxiety; and the dependent variable includes internet addiction. Figure A1 means that the data basically satisfy the multivariate normal distribution and that there are no significant outlier data.
